# Sedentary behavior associated with reduced medial temporal lobe thickness in middle-aged and older adults

**DOI:** 10.1371/journal.pone.0195549

**Published:** 2018-04-12

**Authors:** Prabha Siddarth, Alison C. Burggren, Harris A. Eyre, Gary W. Small, David A. Merrill

**Affiliations:** 1 Semel Institute for Neuroscience and Human Behavior, UCLA, Los Angeles, CA, United States of America; 2 Center for Cognitive Neurosciences, UCLA, Los Angeles, CA, United States of America; 3 Discipline of Psychiatry, University of Adelaide, Adelaide, Australia; Nathan S Kline Institute, UNITED STATES

## Abstract

Atrophy of the medial temporal lobe (MTL) occurs with aging, resulting in impaired episodic memory. Aerobic fitness is positively correlated with total hippocampal volume, a heavily studied memory-critical region within the MTL. However, research on associations between sedentary behavior and MTL subregion integrity is limited. Here we explore associations between thickness of the MTL and its subregions (namely CA1, CA23DG, fusiform gyrus, subiculum, parahippocampal, perirhinal and entorhinal cortex,), physical activity, and sedentary behavior. We assessed 35 non-demented middle-aged and older adults (25 women, 10 men; 45–75 years) using the International Physical Activity Questionnaire for older adults, which quantifies physical activity levels in MET-equivalent units and asks about the average number of hours spent sitting per day. All participants had high resolution MRI scans performed on a Siemens Allegra 3T MRI scanner, which allows for detailed investigation of the MTL. Controlling for age, total MTL thickness correlated inversely with hours of sitting/day (r = -0.37, p = 0.03). In MTL subregion analysis, parahippocampal (r = -0.45, p = 0.007), entorhinal (r = -0.33, p = 0.05) cortical and subiculum (r = -0.36, p = .04) thicknesses correlated inversely with hours of sitting/day. No significant correlations were observed between physical activity levels and MTL thickness. Though preliminary, our results suggest that more sedentary non-demented individuals have less MTL thickness. Future studies should include longitudinal analyses and explore mechanisms, as well as the efficacy of decreasing sedentary behaviors to reverse this association.

## Introduction

Growing evidence from clinical trials, epidemiological and neuroscience research suggests that physical exercise is a promising intervention for delaying the onset of dementia and Alzheimer’s disease [1–4]. Further, physical activity has been shown to have notable beneficial effects on brain structure, both microstructure and macrostructure [5–12]. In contrast to the large literature on physical activity, there is a paucity of research on the relationship between sedentary behavior and dementia risk [13]. This is concerning given that sedentary behaviors may be independent from exercise and other physical activities, and therefore warrant independent inquiry [13–14]. Indeed, one can be highly active yet still be sedentary for most of the day [14]. Several lines of evidence suggest that sedentary behavior may be a risk factor for the development of age-related cognitive impairment [15–16]. A detailed projection of the effect of risk factors on Alzheimer’s disease (AD) prevalence [17] suggests that approximately 13% of AD cases worldwide may be attributable to sedentary behavior. A 25% reduction in sedentary behavior could potentially prevent more than 1 million AD cases globally.

The atrophy and anti-neuroplastic processes occurring in cognitive decline are thought to occur in the medial temporal lobe (MTL). Indeed, global MTL volume atrophy is known to be associated with memory impairment and AD [18]. A growing number of studies have shown that physical activity affects regional brain volumes [7–8, 11], specifically in the hippocampus. However, very few studies have examined the effect of sedentary behavior on brain volumes. A recent study [19] demonstrated an association between a 5-year decrease in white matter volume and increased amount of sedentary behavior in a sample of healthy older adults. A more detailed analysis of the MTL subregional structure, including the hippocampus and neighboring cortical areas, is likely to be important [20]. For example, cortical thickness measures have been used to assess structure changes with memory disorders with reasonable accuracy and reliability, both cross-sectionally [21–22] and longitudinally [23].

Several mechanisms have been postulated for how physical activity improves brain health [24–25], including increased blood flow in the brain to promote the development of new neurons [26] and delaying brain structural and functional decline; in contrast, only few studies [27] have looked at sedentary behavior from a mechanistic perspective. It has been suggested that sedentary behavior may have deleterious effects on glycemic control, and the increased glycemic variability and resultant decreased cerebral blood flow may lead to worse brain health [27].

In this preliminary analysis, we explore associations between physical activity levels, sedentary behavior and thickness in MTL and neighboring cortical sub-region structures in non-demented middle-aged and older adults, examining the relationship of physical activity and time spent sitting simultaneously on brain thickness. We hypothesized that both lower levels of physical activity and time spent sitting will be associated with less thickness in MTL and its subregions.

## Methods

### Participants

Participants were community-dwelling middle-aged and older adults recruited through local advertising, media coverage of the study, and referrals by physicians and families for a longitudinal study of mild memory changes intended to examine brain structure and function using neuroimaging techniques in non-demented individuals (n = 49; [28]). Exclusion criteria included participants with a lifetime history of dementia, major psychiatric or neurologic disorders, alcohol or substance abuse, head trauma or systemic disease affecting brain function, or uncontrolled hypertension or cardiovascular disease. In order to rule out reversible causes of cognitive dysfunction, participants underwent screening evaluations including medical history and physical examination, laboratory tests and an electrocardiogram. Participants were also screened to ensure normal global cognition using the Mini-Mental State Examination (MMSE; at least 28) [29]. All participants also underwent standard clinical and laboratory testing including body mass index (BMI) measurements and APOE genotyping [28]. The Hamilton Rating Scales for both Depression [30] and Anxiety [31] were administered to assess mood and anxiety, respectively. Participants meeting criteria for depressive or anxiety disorders (n = 9) were excluded to control for impact of mood on study measures. Five additional participants, who were younger than 45 years of age, were not included, thus the final sample size was 35 for the analyses. The study procedures were performed at the Semel Institute for Neuroscience and Human Behavior at the University of California, Los Angeles. The study was reviewed and approved by the UCLA Human Subjects Protection Committee and participants gave written informed consent according to the UCLA Human Subjects Protection Committee procedures.

### Neuroimaging and image analysis

All MRI scans were performed on a Siemens Allegra 3T head-only MRI scanner. We acquired sagittal T1-weighted magnetization prepared rapid acquisition gradient-echo (MPRAGE) volumetric scans (TR 2300ms, TE 2.93ms, slice thickness 1mm, 160 slices, inplane voxel size 1.3 *×* 1.3 mm, FOV 256mm) for volumetric measurements and high-resolution oblique coronal T2-weighted fast spin echo (FSE) sequences for structural segmentation and unfolding procedures (TR 5200ms, TE 105ms, slice thickness 3 mm, spacing 0 mm, 19 slices, in-plane voxel size 0.39 *×* 0.39 mm, FOV 200 mm).

**Fig 1:**
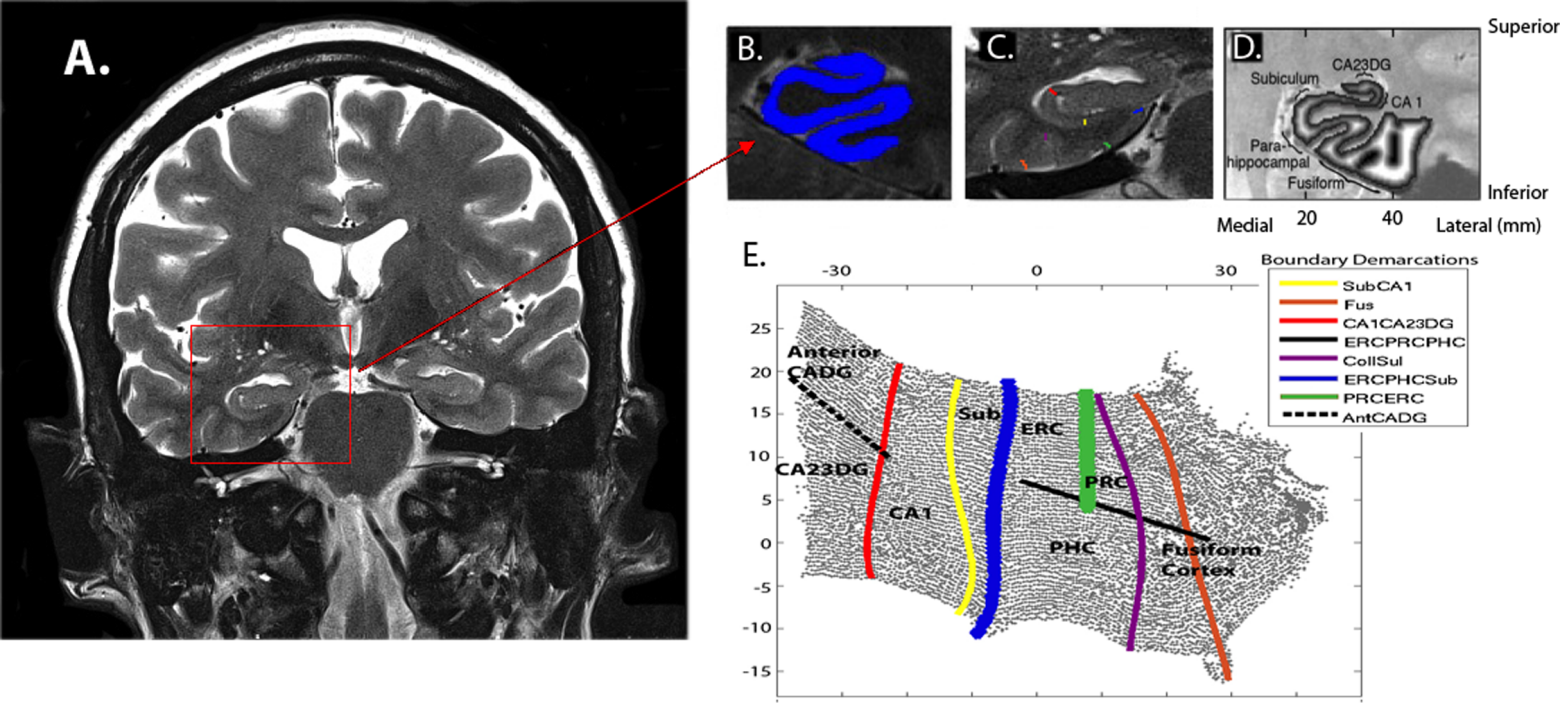
Methods for producing flat maps. Oblique coronal images are acquired to cover the long axis of the hippocampus as shown in image **A**. This image is cropped over the area of interest (red square) and shown in greater detail in image **B**. The gray matter ribbon (shown in bright blue) is segmented out from the surrounding MTL area. **C**. Boundaries between subregions are demarcated on each slice and projected to the corresponding location in flat-map space (shown in E). Boundaries between these subregions are shown in C and projected to flat-map (or 2D) space as shown in Fig E. **D**. Thickness in gray matter space is calculated in-plane space by taking the maximum distance value of the corresponding 3D voxels across all layers and multiplying by two to arrive at a thickness value for each voxel. **E**. The subregions are labeled as follows: cornu ammonis (CA) fields 1, 2, 3 and the dentate gyrus (DG) subiculum (Sub), entorhinal cortex (ERC), perirhinal cortex (PRC), parahippocampal cortex (PHC) and fusiform cortex (Fus). Boundary colors in **C** correspond to the same color scale in **E** and are labeled according to the *Boundary Demarcation* color bar.

We used cortical unfolding [21–22] to enhance the visibility of the convoluted MTL cortex by flattening the entire MTL gray matter volume to 2D-space **(Fig 1)**. First, we manually defined white matter and cerebrospinal fluid (CSF) on the oblique coronal T2 FSE structural MRI sequence with high in-plane resolution. In order to maximize visibility of the images for manual segmentation, high in-plane resolution (0.39 *×* 0.39mm) is critical. To minimize the effect of this larger through-plane resolution across slices on boundary changes, we acquired images perpendicular to the long axis of the hippocampus (HC) where anatomical variability in HC structures is smallest, thereby minimizing variability from slice to slice while maximizing in-plane resolution where anatomic variability is greatest. Once segmentation is complete, the original images are interpolated by a factor of 7, resulting in a final voxel size of 0.39 *×* 0.39 *×* 0.43 mm. Next, up to 18 connected layers of gray matter are grown out from the boundary of white matter, using a region-expansion algorithm to cover all pixels defined as gray matter. This produces a gray matter strip containing cornu ammonis (CA) fields 1, 2, and 3, the dentate gyrus (DG), subiculum (Sub), entorhinal cortex (ERC), perirhinal cortex (PRC), parahippocampal cortex (PHC), and the fusiform gyrus (FUS). We are unable to distinguish between CA fields 2, 3, and DG due to limits in resolution; thus we treat these regions as a single entity (CA23DG). It is this strip of gray matter that is the input for the unfolding procedure, an iterative algorithm based on multidimensional scaling (http://www.ccn.ucla.edu/wiki/index.php/Unfolding). We delineated boundaries between subregions on the original in-plane MRI images, based on histological and MRI atlases and then projected them mathematically to their corresponding coordinates in flat map space. We calculated cortical thickness in all MTL subregions (CA23DG, CA1, SUB, ERC, PRC, PHC, and FUS), averaged over left and right hemispheres, as well as total MTL cortical thickness by averaging thickness across these subregions. To calculate thickness, for each gray matter voxel we computed the distance to the closest non-gray matter voxel. In 2D-space, for each voxel, we took the maximum distance value of the corresponding 3D voxels across all layers and multiplied by two. Mean thickness in each subregion was calculated by averaging thickness of all 2D voxels within each region of interest.

Manual segmentations were finalized and readied for unfolding procedures by the same person. This investigator was unaware of all demographic and clinical information. All manual segmentations were performed in native space, in line with previous studies using the cortical unfolding technique. We have previously reported interrater and test-retest reliability analyses for the manual procedures involved [32].

We also used a whole brain voxel-based approach to examine regions of significant differences by physical activity levels. Whole brain T1 scans were preprocessed using Freesufer v6.0 qdec (https://surfer.nmr.mgh.harvard.edu/fswiki/recon-all). All structural outputs were visually inspected for proper cortical segmentation before inclusion in any analyses.

### Assessment of physical activity levels and time spent sitting

Level of physical activity and time spent sitting were determined using the self-reported International Physical Activity Questionnaire modified for older adults (IPAQ-E), which is validated for use in non-demented middle-aged and older adults [33–35].The IPAQ-E consists of 4 sets of questions assessing walking, moderate physical activities, vigorous physical activities, and average time spent sitting per day. Total physical activity is quantified by weighting each type of activity by its energy requirements defined in metabolic equivalent units (METs) to yield a score in MET-minutes per week. For the current analyses, physical activity was examined both as a continuous variable and as a categorical variable: participants were dichotomized into either “lower” or “higher” activity groups based on a standard metabolic equivalent (MET) cutoff of 1500 MET-minutes per week. This cutoff was chosen as a mid-point between no activity (0 METs) and high activity (3000 METs), as defined on the IPAQ website (www.ipaq.ki.se). Sitting data from the IPAQ-E was reported as average number of hours spent sitting per weekday over the past week. Weekend days were not included as prior work has shown lower accuracy of self-reported sitting for weekend days [36].

### Statistical analysis

Prior to analyses, all data were inspected for outliers, skewness, and homogeneity of variance to ensure their appropriateness for parametric statistical tests. Due to the skewed distribution of physical activity levels, we used log-transformed values in all analyses using continuous physical activity measures. Descriptive statistics were compiled for all demographic data and study variables. We first conducted exploratory univariate analyses examining if MTL thickness (total as well as subregional) were related to age, sex, BMI, education, ethnicity, and APOE-4 status, using Pearson’s correlations for continuous and t-tests for binary measures. Only those variables that were associated with a p ≤ 0.1 in the univariate analyses were retained for further modeling. A general linear model was used to determine if total MTL thickness was significantly associated with physical activity (either continuous or defined as lower vs. higher) and time spent sitting, controlling for those covariates identified above. We also examined interaction terms between both physical activity and sitting with the potential covariates. If either of the two predictors of interest (physical activity or sitting) was found to have a significant effect on total MTL thickness, follow-up analyses were conducted to examine which subregions within the MTL contributed to the significant finding. To correct for inflation of Type I error, we used the Benjamini-Hochberg correction for false discovery rate to adjust for multiple comparisons. For significant associations, findings are also presented as adjusted Pearson correlation coefficients (r). A significance level of p ≤ 0.05 (two-tailed) was used for all inferences.

As a sensitivity analysis, we examined regions of significant differences by physical activity levels using the whole brain voxel-based approach. Two models were set up using FreeSurfer: (1) Does the correlation between thickness and time spent sitting differ from zero (using sitting hours as a continuous covariate of interest, including nuisance covariates for age, sex, BMI, education, ethnicity, and APOE-4 status)? (2) Does the average thickness differ between low and high physical activity (using physical activity groups (low and high) as a discrete (fixed) factor, including the same nuisance covariates as above)? Dependent variables used for both analyses included surface-based and morphometric thickness measures, using a smoothing parameter of 20 full-width at half-maximum (FWHM). A “Different Offset, Different Slope” (DODS) design matrix type was chosen for both analyses. A False Discovery Rate (FDR) threshold of 0.05 was applied to address the multiple comparisons issue. A Monte Carlo Null-Z Distribution (threshold = 2.0 (p = 0.01), Sign = absolute) was also applied to the analyses to correct for multiple comparisons.

## Results

### Demographics and sample characteristics

Participants (n = 35; 82.9% Caucasian) were middle-aged and older adults (25 women, 10 men) with an age range of 45 to 75 years (mean ± SD = 60.4 ± 8.1); education in the sample averaged 16.4 ± 2.5 years **(Table 1)**. BMI of the sample ranged from 19 to 35 (25.4 ± 3.6), with 15 participants having a BMI of over 25. MMSE and the other cognitive scores for all participants were within expected range and indicated that all participants were cognitively normal. The scores for the Hamilton Rating Scales for Depression and Anxiety averaged 1.8 ± 3.0 (SD) and 4.3 ± 3.5 (SD), respectively. There were 15 APOE-4 carriers and 20 non-carriers.

**Table 1:** Demographic, clinical and physical activity characteristics of study sample.

Characteristic*	Sample (n = 35)
Age	60.4 (8.1)
Sex—Female	25 (71.4%)
Race—Caucasian	29 (82.9%)
Education (years)	16.4 (2.5)
BMI	25.4 (3.6)
APOE-4	15 (42.9%)
MMSE	29.3 (0.7)
Digit Symbol^$^	66.9 (16.2)
Verbal Paired Associations^¶^	6.6 (2.2)
Selective Reminding^	8.0 (3.3)
HAM-D	1.8 (3.0)
HAM-A	4.3 (3.5)
Time sitting (hours/day)	7.2 (3.3)
Physical activity (MET minutes per week)	1521 (1225)

*Results are reported as mean (SD) for continuous variables and number of participants (%) for categorical variables.

Abbreviations: BMI = Body mass index; APOE-4 = Apolipoprotein E-4; MMSE = Mini-mental status exam; HAM-D = Hamilton depression scale; HAM-A = Hamilton anxiety inventory; MET = Metabolic equivalent units

^$^ Scores range from 49 to 72, with higher scores indicating better functioning.

^¶^ Scores range from 0 to 8, with higher scores indicating better cognitive functioning.

^Scores range from 0 to 12, with higher scores indicating better cognitive functioning.

Physical activity levels averaged 1521 ± 1225 MET minutes per week, and 21 individuals had lower levels whereas 14 participants had higher levels (using a standardized cutoff of 1500 MET-minutes per week); time spent sitting averaged 7.2 ± 3.3 hours/day for all participants. Time spent sitting was not related to physical activity levels (r = 0.03, p = 0.9) or groups (t(33) = 0.8, p = 0.4).

### Relationship between MTL thickness measures, physical activity levels, and sitting

**Fig 2:**
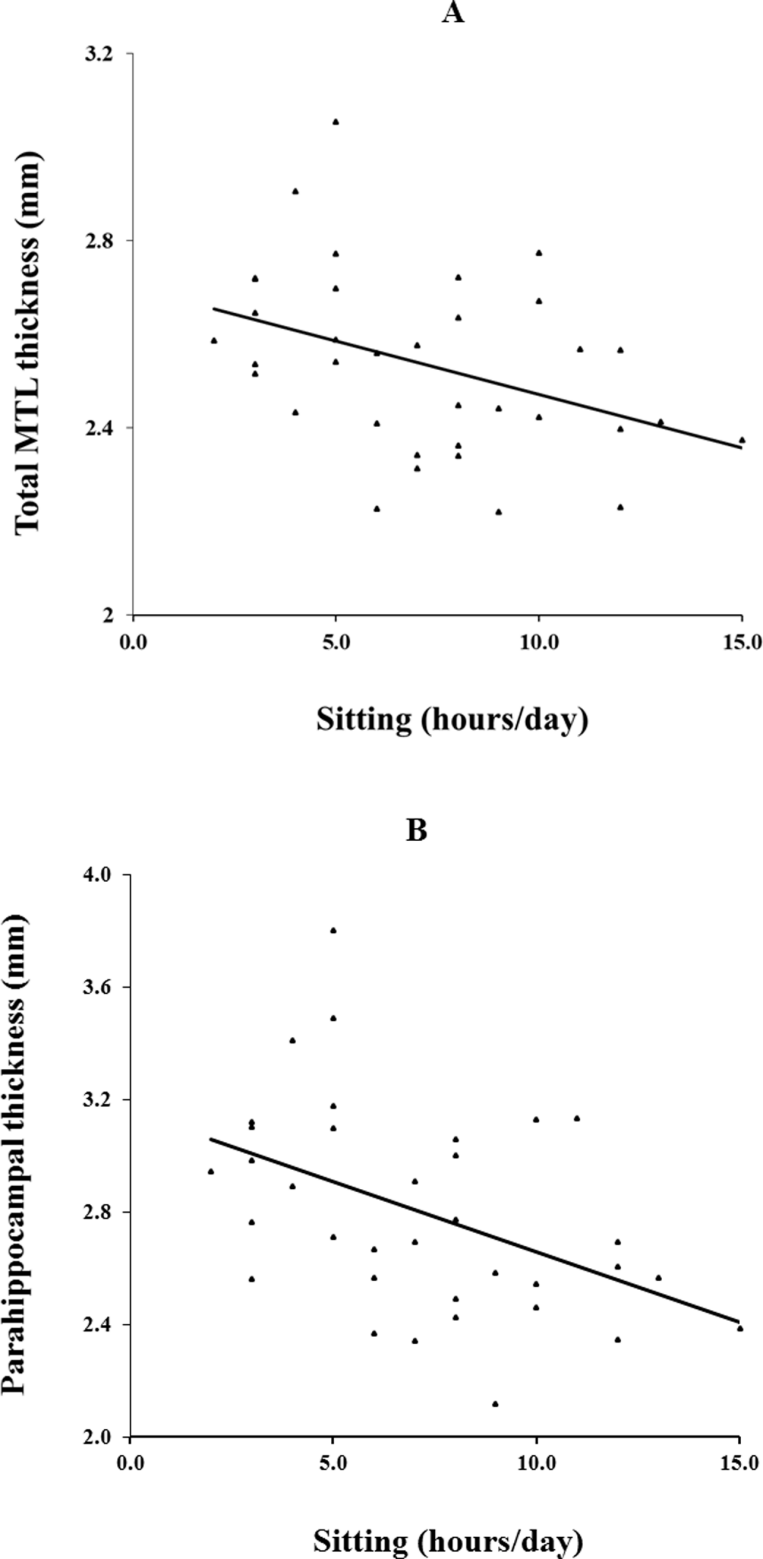
Correlation of average number of hours spent sitting per day to thickness. (A) Total medial temporal lobe (MTL) (r = -0.37, p = .03) and (B) parahippocampal (r = -0.45, p = .007) thickness correlated inversely with hours of sitting/day, controlling for age.

Only age was associated (p ≤ .1) with total and subregional MTL thickness, and was therefore included as a covariate in the general linear models. These models thus included time spent sitting, physical activity (either as a continuous or categorical variable) and age as predictors. None of the interaction terms (between age and physical activity; age and sitting time) were found to be significant. A significant negative association was found between hours of sitting in a day and total MTL thickness (F(1,31) = 5.0, p = .03) **(Table 2)**. In contrast, physical activity, whether entered into the model as a continuous (F(1,31) = 0.04, p = .8)) or as a categorical (F(1,31) = 0.1, p = .7)) measure was not associated with thickness. Region-specific analyses revealed that thicknesses of the entorhinal cortex (F(1,31) = 4.0, p = .05), parahippocampal cortex (F(1,31) = 8.3, p = .007), and subiculum (F(1,31) = 4.5, p = .04) were significantly associated with time spent sitting. All these associations survive the Benjamini-Hochberg correction for false discovery rate. As with total MTL thickness, physical activity was not associated with any of the subregional thicknesses. Partial correlations (adjusting for age) between time spent sitting and MTL thicknesses, for those regions with significant associations, are: Total: r = -0.37, p = .03; entorhinal cortex: r = -0.33, p = .05; parahippocampal cortex: r = -0.45, p = .007; and subiculum: r = -0.36, p = .04 **(Fig 2)**.

**Table 2:** Predictors of total and regional medial temporal lobe (MTL) thickness.

Model	Predictor	β (95% CI)	p-value
Total MTL thickness	Sitting	***-0*.*02 (-0*.*04*, *-0*.*002)***	***0*.*03***
	Physical activity*	0.007 (-0.07, 0.08)	0.8
CA1 thickness	Sitting	-0.004 (-0.02, 0.008)	0.5
	Physical activity*	-0.02 (-0.06,0.03)	0.5
CA23DG thickness	Sitting	-0.007 (-0.03, 0.01)	0.5
	Physical activity*	0.05 (-0.02, 0.13)	0.2
ERC thickness	Sitting	***-0*.*03 (-0*.*07*, *-0*.*001)***	***0*.*05***
	Physical activity*	-0.03 (-0.16, 0.10)	0.6
FUS thickness	Sitting	-0.01 (-0.03, 0.006)	0.2
	Physical activity*	0.02 (-0.06, 0.09)	0.7
PHC thickness	Sitting	***-0*.*05 (-0*.*08*, *-0*.*01)***	***0*.*007***
	Physical activity*	0.03 (-0.11, 0.17)	0.7
PRC thickness	Sitting	-0.03 (-0.06, 0.004)	0.09
	Physical activity*	-0.07 (-0.18, 0.04)	0.2
SUB thickness	Sitting	***-0*.*02(-0*.*05*, *-0*.*001)***	***0*.*04***
	Physical activity*	0.05 (-0.04, 0.13)	0.3

*Reported statistics are for physical activity modeled as log-transformed continuous variable; for all models, age was included as a covariate.

Abbreviations: CA1 = Cornu Ammonis field 1; CA23DG = Cornu Ammonis fields 2,3 and Dentate Gyrus; ERC = Entorhinal Cortex; FUS = Fusiform Gyrus; MTL = medial temporal lobe; PHC Parahippocampal Cortex; PRC = Perirhinal Cortex; SUB = Subiculum.

In addition, the results of the fully adjusted model (including age, sex, BMI and education as covariates) with sitting time and physical activity as predictors of total MTL thickness indicate that the only predictor that approached significance was time spent sitting (β = -0.02, p = .07); as with the reduced model, physical activity was not significant (β = 0.01, p = .8). Further, the whole brain voxel-based structural analyses found no regions of significant differences by physical activity levels or time spent sitting.

## Discussion

In this neuroimaging study examining relationships between self-reported sedentary behavior, physical activity and thickness of MTL, we found that sedentary behavior, but not physical activity, was associated with less thickness in the MTL and its subregions (parahippocampal cortex, entorhinal cortex and subiculum). MTL structures are essential to memory function [37–38] and the hippocampal formation and entorhinal cortex are particularly affected by neuropathological findings very early in the course of AD, prior to dementia [39–40]. A review suggests that the parahippocampal and medial entorhinal cortex are in particular essential to spatial recognition [37]. Thus, the finding that more sedentary time is associated with less thickness in MTL is clinically relevant and suggests that reducing this behavior may be a possible target for interventions designed to improve brain health in middle-aged and older adults.

While previous studies have shown that physical activity is related to brain imaging measures such as total brain and hippocampal volumes, we did not find a significant association of physical activity and MTL thickness in this sample. This study explores the associations of physical activity and time spent sitting *simultaneously* on brain thickness. Hence it is possible that sedentary behavior is a more significant predictor of brain structure, specifically MTL thickness, and that physical activity, even at higher levels, is not sufficient to offset the harmful effects of sitting for extended periods of time. Indeed, it has been suggested recently [15] that reducing sedentary behavior may reduce glycemic variability, protecting against cognitive decline and this lifestyle change may be an additional strategy, in addition to increasing physical activity, especially in older adults. In fact, increasing evidence suggests that intervention studies aimed at improving subsequent health outcomes in older adults may be more effective if reducing sedentary behavior is the more immediate goal [41].

The voxel-based morphometric analysis did not reveal significant relationships between volumetric brain data and either time spent sitting or PA. However, we [21–22], and others [38, 42], have demonstrated that age-related differences in regional brain volume measurements may reflect differences in underlying pathological changes related to either normal aging or disease-related processes. In fact, assessing volume of parcellated cortical regions, as in the voxel-based morphometric analysis, is a composite measure related to both surface area and thickness. It is not clear whether volumetric decreases in medial temporal lobe cortical regions in normal aging or age-related diseases (such as AD) are due to thinning, loss of surface area, or both, nor is it clear whether aging and AD differ in their effects on these properties. However, previous results [21] indicate that assessments of cortical thickness are more sensitive to structural brain changes in cognitively intact older adults. Structural changes in these subjects are likely to be much more subtle than the gross morphological brain changes seen in clinically-diagnosed Alzheimer's disease patients and hence are more likely to be observed with cortical thickness related measures than voxel-based morphology methods.

To date, no studies have explored associations between sedentary behaviors and MTL subregion structure. A previous study [43] has explored the association between HC physiology (blood flow) and sedentary time in healthy older adults. These authors found that the relationship between sedentary time and cerebral blood flow in the left hippocampus differs by APOE-4 status, whereby APOE-4 carriers show higher cerebral blood flow as a function of longer sedentary time compared to non-carriers, possibly suggesting a cerebral blood flow regulatory response to compensate for metabolic alterations in dementia risk i.e. blood flow increases due to increased demand for glucose and oxygen to support neuronal activity. In our study we did not find an effect of APOE-4 status to thickness in the MTL.

The mechanism of how sedentary behavior is associated with thickness in MTL regions is uncertain. Sedentary behaviors appear to have direct effects on neurobiological processes. A review [36] outlines evidence to suggest that sedentary behavior may have detrimental effects on the brain via reducing neurogenesis, synaptic plasticity, neurotrophin production, angiogenesis, and by increasing inflammation, all pathological processes known to affect hippocampal integrity. Sedentary behavior is also associated with increased cardiovascular and metabolic risk factors, such as diabetes, hypertension, and obesity [44] and impaired vascular supply may also play a role. Studies such as this one, indicating associations between sedentary time and neuroimaging measures, are an important first step to determining the pathways by which physical activity and sedentary behaviors affect the brain.

We are aware of only one rodent study exploring the effects of physical activity cessation on HC neuroplasticity [45]; in general, the findings are concordant with our study. In this study, male C57BL/6 mice of 4 weeks old were exposed to a variety of conditions until 21 weeks old to allow for an assessment of the effects of exercise cessation. The marker of neurogenesis (BrdU-labelled cells) in the dentate gyrus was equivalent between mice exposed to no exercise vs. mice exposed to exercise for 8 weeks, then changed to no exercise. Another marker of neurogenesis (BrdU-positive cells to doublecortin-positive immature neurons) was significantly lower in the exercise cessation group vs. all other groups. This data suggests exercise cessation has anti-neuroplastic effects in the dentate gyrus. Assessing the direct effects of sedentary behavior on rodent models will allow for a more detailed understanding of mechanisms.

This preliminary analysis of subjectively reported physical activity and sedentary time has limitations. This study utilized a questionnaire to explore physical activity and sedentary behavior. While such as measure is simple and easy to use, it has limitations, especially in older adults with difficulty recalling past events. However, it should be noted that all MMSE scores were normal in our sample, and no participants had functional deficits. This study drew from a convenience sample of non-demented middle-aged and older adults. An important extension of this study would be to explore MTL structures in healthy younger adults, as an example of universal or primary prevention as opposed to our secondary prevention study of those at-risk individuals with minimally detectable symptoms. Another key limitation is the small sample size, and it is possible that we did not have sufficient power to detect the association of physical activity or the interaction terms. We also did not have variables such as hypertension, smoking and alcohol consumption that may have an effect on thickness of brain structures. Finally, this analysis was cross-sectional and therefore causation cannot be assessed. A longitudinal assessment is important to allow for some conclusions regarding causation and/or time course of observed changes. Strengths of this study include the high resolution MRI scanning, which allows for a detailed assessment of MTL structures as well as simultaneously examining the associations of sedentary behavior and physical activity on MTL structures. A wide range of covariates, including age, sex, education, ethnicity, BMI, and APOE-4 status was also evaluated.

The findings of the present study, while novel and with important implications, raise several unanswered questions. Exploring the role of biological mechanisms as mediators for the effects of sedentary behavior on neuroplasticity markers is a key consideration (e.g. serum immune biomarker assessment). There may also be differences in the effect of various types of ‘sitting’ behaviors, and this is an area for further investigation. It is possible that there may be two distinct groups: mentally active sitting and mentally inactive sitting. In mentally active sitting, individuals may be attending to cognitive demanding tasks such as crossword puzzles, documentation, writing, or computer games. In mentally inactive sitting, individuals may be engaging in less demanding, passive tasks such as watching television or movies. Other lifestyle factors such as dietary patterns may also impact these findings and so should be explored and controlled for in future studies.

## Conclusions

In this preliminary study of middle-aged and older adults, self-reported hours per day spent sitting, but not physical activity level, was associated with less thickness in the MTL substructures. These findings are novel and require further exploration in longitudinal studies and analysis of mediating mechanisms. Better understanding the effects of sedentary behavior on our brains is important given the global epidemic of physical inactivity and sedentary lifestyles.

## Supporting information

S1 Table Demographic variables, physical activity, cognitive scores and brain thickness measurements of all participants. (XLSX)
